# Nano-Pulse Stimulation induces immunogenic cell death in human papillomavirus-transformed tumors and initiates an adaptive immune response

**DOI:** 10.1371/journal.pone.0191311

**Published:** 2018-01-11

**Authors:** Joseph G. Skeate, Diane M. Da Silva, Elena Chavez-Juan, Snjezana Anand, Richard Nuccitelli, W. Martin Kast

**Affiliations:** 1 Department of Molecular Microbiology & Immunology, University of Southern California, Los Angeles, CA, United States of America; 2 Department of Obstetrics & Gynecology, University of Southern California, Los Angeles, CA, United States of America; 3 Norris Comprehensive Cancer Center, University of Southern California, Los Angeles, CA, United States of America; 4 Pulse Biosciences, Inc., Hayward, CA, United States of America; Istituto Superiore Di Sanita, ITALY

## Abstract

Nano-Pulse Stimulation (NPS) is a non-thermal pulsed electric field modality that has been shown to have cancer therapeutic effects. Here we applied NPS treatment to the human papillomavirus type 16 (HPV 16)-transformed C3.43 mouse tumor cell model and showed that it is effective at eliminating primary tumors through the induction of immunogenic cell death while subsequently increasing the number of tumor-infiltrating lymphocytes within the tumor microenvironment. *In vitro* NPS treatment of C3.43 cells resulted in a doubling of activated caspase 3/7 along with the translocation of phosphatidylserine (PS) to the outer leaflet of the plasma membrane, indicating programmed cell death activity. Tumor-bearing mice receiving standard NPS treatment showed an initial decrease in tumor volume followed by clearing of tumors in most mice, and a significant increase in overall survival. Intra-tumor analysis of mice that were unable to clear tumors showed an inverse correlation between the number of tumor infiltrating lymphocytes and the size of the tumor. Approximately half of the mice that cleared established tumors were protected against tumor re-challenge on the opposite flank. Selective depletion of CD8^+^ T cells eliminated this protection, suggesting that NPS treatment induces an adaptive immune response generating CD8^+^ T cells that recognize tumor antigen(s) associated with the C3.43 tumor model. This method may be utilized in the future to not only ablate primary tumors, but also to induce an anti-tumor response driven by effector CD8^+^ T cells capable of protecting individuals from disease recurrence.

## Introduction

Human papillomavirus (HPV)-associated anogenital and head and neck cancers cause significant morbidity and mortality worldwide. In general, HPV is detected in more than 90% of anal and cervical cancers, ~70% of oropharyngeal, vulvar, and vaginal cancers, and greater than 60% of penile cancers [1]. Cervical cancer is the fourth most frequent cancer among women in the world. More specifically there are over 520,000 newly diagnosed cervical cancers each year, and worldwide cervical cancer mortality is 6.8 per 100,000 women [2]. Although effective prophylactic HPV vaccines aimed at targeting the L1 capsid protein have been developed and approved for use [3, 4], uptake of vaccination has been slow and does not show therapeutic efficacy for individuals already infected with a high-risk HPV genotype or those harboring an HPV-transformed tumor [5, 6]. Because HPV-transformed cancers are expected to continue their upward trajectory in the foreseeable future, an effective therapy that leads to the generation of anti-HPV T cell immunity may provide a novel method to treat established cancers.

Nano-Pulse Stimulation (NPS) is a new non-thermal tumor treatment modality that uses ultra-short electric pulses to induce immunogenic cell death in treated tissues. To date NPS has been used to treat non-viral tumor types, and the results have shown target cells undergo immunogenic cell death that then leads to necrosis and slow regression over a period of weeks [7–10]. After NPS treatment, cells of the innate immune system are recruited to the treated tumor and phagocytose tumor cells. Within 3 weeks, CD8^+^ cytotoxic T cells are generated that target those tumor cells [11–13]. In order to determine if NPS could be effective at eliminating viral-driven cancers, we examine within this manuscript the *in vitro* treatment effects of NPS on the HPV16-transformed murine tumor cell line, C3.43, with respect to caspase activation and phosphatidylserine (PS) translocation to the outer leaflet of the plasma membrane as well as *in vivo* treatment effects on established subdermal C3.43-tumors in immunocompetent mice.

## Materials and methods

### Mice, cell lines, and antibodies

The study was carried out in strict accordance with the recommendations in the Guide for the Care and Use of Laboratory Animals of the National Institutes of Health. Specifically, mice were monitored by USC veterinary staff daily and euthanization was carried out immediately if mice were bearing tumors greater than 10% of bodyweight, measured >1500 mm^3^, or if outer tumor epithelium had been compromised by ulceration. During this study we did not have any animals that died outside of these conditions. All NPS treatments were carried out while mice were anesthetized under continuous 2% isoflurane in oxygen. The protocol and all procedures were approved by the University of Southern California Institutional Animal Care and Use Committee (Permit number 20065). Specific pathogen-free female C57BL/6 (B6) mice, 6 to 8 weeks old, were purchased from Taconic Farms. Tumor challenge studies were performed using the C3.43 cell line, an *in vivo* passaged derivative of the C3 HPV16-transformed B6 murine tumor cell line [14]. C3.43 cells have retained expression of the HPV16 E6 and E7 under the natural HPV promotor, express similar levels of MHC class I molecules on the surface compared with the parental C3 line, and respond to prophylactic vaccination with HPV16 E7–containing vaccines *in vivo* [15]. C3.43 cells tested negative for Mycoplasma contamination (MycoAlert Mycoplasma Detection kit, Lonza, Walkersville, MD). Cells used for tumor challenge were cultured for 10–11 days from seed stocks in Iscove's modified Dulbecco's medium supplemented with 10% fetal bovine serum before *in vivo* challenge. The following phenotyping antibodies were purchased from BioLegend (San Diego, CA): CD3 FITC (clone 145-2C11), CD4 PE-Cy5.5 (clone GK 1.5), CD8a PE-Cy7 (clone 53–6.7), CD45 APC-Cy7 (clone 30-F11), rat IgG2a FITC, rat IgG1 PE-Cy5, rat IgG2b PE-Cy7, and rat IgG2b APC-Cy7.

### Annexin V/7-AAD apoptosis detection assay

The PE Annexin V Apoptosis Detection Kit I (BD Biosciences) was used to assay the percentage of cells undergoing the stages of apoptotic cell death. Cells were treated with NPS (as described below) and then incubated at 37°C with 5% CO_2_. Cells were harvested at 1, 3 and 24 h post treatment. After harvesting, cells were washed twice with 1X PBS (wash: suspend in 100 μl buffer; centrifuged at 1200 rpm for 5-minutes at 4°C) followed by resuspension in 100 μl of Annexin V/7-AAD (7-Aminoactinomycin D) staining cocktail (1 μl PE Annexin V and 1 μl 7-AAD in 100 μl 1X Annexin Binding Buffer). Cells were protected from light and incubated for 15-minutes at room temperature. After incubation, 100 μl binding buffer was added to each sample and gently mixed.

Stained cells were analyzed on a Beckman CytoFLEX flow cytometer. Cells were gated based upon Annexin V binding (PE Annexin V: Ex 488/Em 578) and cell viability (7-AAD: Ex 488/Em 647). Gated cells were binned into 4-populations based upon stage of cell death: live viable cells (PE Annexin V-/7-AAD-); early apoptotic (still viable) (PE Annexin V+/-AAD-); late stage apoptotic/necrotic (non-viable) (PE Annexin V+/7-AAD+); very late stage cell death (non-viable) (PE Annexin V-/7-AAD+). Binned populations were expressed as % of total cells.

### Activated caspase assay

Activation of combined caspase-3 and caspase-7 was assessed using the Caspase-Glo^®^ 3/7 Assay (Promega). Following NPS treatments, 1.5x10^4^ C3.43 cells were plated in triplicates within a 96-well assay plate containing pre-equilibrated media and incubated for 3hrs at 37° C, and 5% CO_2_. Caspase-Glo reagent was added to each well at a ratio of 1:1 with cell culture media. This reagent lyses cells and contains pro-luminescent caspase-3/7 substrate, which contains the tetrapeptide sequence, DEVD. This substrate is cleaved to release amino-luciferin, a substrate of luciferase used in the production of light. Caspase is released from the lysed cells and cleaves the substrate to generate the luminescence signal. Samples were incubated for an additional 30 min at room temperature, protected from light, and gently agitated. Sample luminescence was then measured using the Molecular Devices SpectraMax i3 plate reader. Caspase activation was assessed by raw luminescence units (RLUs) of pulsed samples and statistical comparisons were made against the untreated group.

### Pulse generator

All treatments were applied with a 100-ns pulse generator (Transient Plasma Systems, Torrance CA) that was tuned to deliver a relatively square pulse into a 200-ohm load using magnetic compression technology. Optimized treatments were applied at 3 pulses per second (pps). The typical pulse rise time was 25 ns and a typical pulse delivered 65–80 A of current at 30 kV/cm, delivering approximately 0.1 J of energy into the tumor 3 times per second, delivering 0.3 watts.

### Tumor challenge and Nano-Pulse Stimulation (NPS) treatment

**Fig 1:**
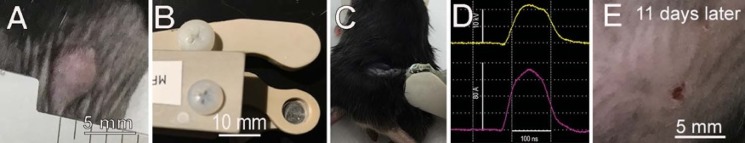
NPS application. **(A)** Photo of a typical shaved C3.43 tumor prior to treatment. **(B)** Pinch electrode used to treat these tumors. **(C)** Pinch electrode sandwiching a tumor as NPS is applied. **(D)** Oscilloscope trace of voltage (top) and current (bottom) applied to the tumor in each pulse. **(E)** Photo of the treated tumor in “A” 11 days later.

Groups of 10 to 15 eight-week-old female C57BL/6 mice were challenged s.c. in the right flank with 5×10^5^ C3.43 tumor cells suspended in 100 μl HBSS. Ten days after tumor cell injection, once tumors had grown to a mean diameter of 3–5 mm (**Fig 1A**), groups receiving NPS were treated. 600 pulses 100 ns-long and 30 kV/cm in amplitude were applied at 3 pps using an electrode **(Fig 1B)** that sandwiched the tumor between two flat cylindrical polished stainless-steel plates 6 mm wide with a spacing of 3 mm between the two plates (**Fig 1C**). The pulses (**Fig 1D**) were applied 300 pulses at a time and the electrode was repositioned over the tumor between applications to ensure coverage of the entire tumor. Most tumors shrunk away completely within 11 days following NPS treatment with light scarring (**Fig 1D**). Throughout the duration of the experiments tumor growth and overall survival was assessed. Tumor size was measured two to three times per week via caliper and volume (mm^3^) was calculated based on L×W×H. Mice were euthanized when tumor volume exceeded 1,500 mm^3^ or if ulceration occurred according to experimental humane endpoints. Mice euthanized were marked as dead the following day for the survival analysis. For tumor re-challenge experiments, mice were given 5×10^5^ C3.43 cells s.c. on the opposite side of the primary tumor.

### Tumor-infiltrating lymphocytes (TIL) isolation and flow cytometry phenotyping

Tumors were isolated from individual mice and processed into single cell suspensions using a mouse tumor dissociation kit with the GentleMACS system (Miltenyi, Auburn, CA) according to manufacturer’s instructions. Cell suspension was passed through a 70 μm nylon strainer to generate a single cell population and TIL separated from tumor cells and debris via a Lympholyte-M gradient (Cedarlane, Burlington, NC). Isolated TILs were incubated with 1:200 dilution of Zombie Aqua (Biolegend, San Diego, CA) to stain for dead cells, washed twice with PBS, incubated with Fc block (Biolegend) for 30 minutes on ice, and then stained for surface antigens indicated by flow panel for 1 hour at 4°C. After washing, cells were fixed with FluoroFix buffer containing 1% paraformaldehyde (Biolegend), washed, and analyzed via flow cytometry. A minimum of 20,000 CD45^+^ events were acquired on the BD FACSCanto II. Flow data were analyzed utilizing FlowJo software (ver. 10.3). Populations were first gated on viable cells using a Zombie Aqua live/dead indicator dye, and CD45^+^ to indicate lymphocyte population. The following sub-gate markers were used for specific populations: CD3^+^CD4^+^ (CD4 T cells), CD3^+^CD8^+^ (CD8 T cells).

### CD4 and CD8 T cell ablation assays

Groups of 10–12 C57Bl/6 mice were challenged with 1.0×10^5^ C3.43 tumor cells and NPS treated as described above. Post NPS treatment, mice were randomized into three groups. Mice that cleared tumors through NPS treatment were then subjected to selective CD4 or CD8 depletion 28-days post initial tumor challenge and 3-days before tumor re-challenge as indicated. Depletion was carried out by 3 consecutive daily IP doses of 500 μg α-CD4 (clone GK1.5) or α-CD8 (clone 2.43) antibodies followed by maintenance dosing every 3^rd^ consecutive day for the duration of the experiment. Depletion was confirmed by weekly flow cytometry analysis of circulating cells collected through retro orbital bleeds (data not shown): flow analysis was based on surface expression of CD3^+^CD4^+^ (CD4 T cells), CD3^+^CD8^+^ (CD8 T cells). All groups were re-challenged at day 31, including a 4^th^ group of age-matched naïve mice to control for tumor take. Tumor growth and survival was monitored as described above.

### IFNγ Enzyme Linked Immunospot (ELISpot) assay

96-well ELISpot plates (Millipore Multiscreen HTS IP) were coated with 5 μg/ml IFNγ capture Ab (Clone AN18, BD Biosciences) in sterile PBS overnight at 4°C. Plates were washed twice with sterile PBS. Complete RMPI medium was then used to block plates for 2h at 37°C. Splenocytes isolated from treated mice were plated in triplicate at 5x10^5^ cells per well in regular medium or medium containing a final concentration of either 2 μg/ml of HPV16 E7_49-57_ peptide [14], 2 μg/ml of ampitope peptide [16], or 1 μg/ml of Concanavalin A (Sigma Chemical Co.). After 20h of incubation at 37°C, plates were washed six times with 0.05% PBST and were incubated with 1 μg/ml of biotinylated IFNγ antibody (Clone R4-6A2, BD Biosciences) in PBS containing 0.5% BSA for 2h at room temperature. Plates were washed six times with 0.05% PBST and wells were subsequently incubated with 100 μl of 1:4000 diluted streptavidin-horseradish peroxidase (Sigma Chemical Co.) for 1h at room temperature. Spots were developed using an AEC (3-amino-9-ethyl-carbazole) (Sigma Chemical Co.) substrate for 5 min and reactions were quenched with deionized water. A Zeiss KS ELISPOT microscope was used to determine the number of spots per well. HPV16 E7_49-57_ specific T cells were quantified after subtraction of background spots from medium control wells. For control responses to known antigens of the C3.43 tumor model in ELISpot experiments, mice were vaccinated with HPV16-Venezuelan Equine Encephalitis replicon particles (VRP) [17], Specifically, 5 age-matched, non-tumor-bearing mice were vaccinated with 1.0×10^7^ infectious units of VRP i.m. in 50 μL PBS on days 14 and 21 and utilized as positive controls for responses against the E7_49-57_ peptide.

### Statistical analysis

All statistical analyses were performed on GraphPad Prism version 6.0 (GraphPad Software Inc., San Diego, CA). For each statistical method used an alpha of 0.05 was used as our measure of significance. Methods to correct for multiple comparisons were utilized when three or more groups were analyzed.

## Results

### NPS triggers immunogenic cell death in C3.43 cells

**Fig 2:**
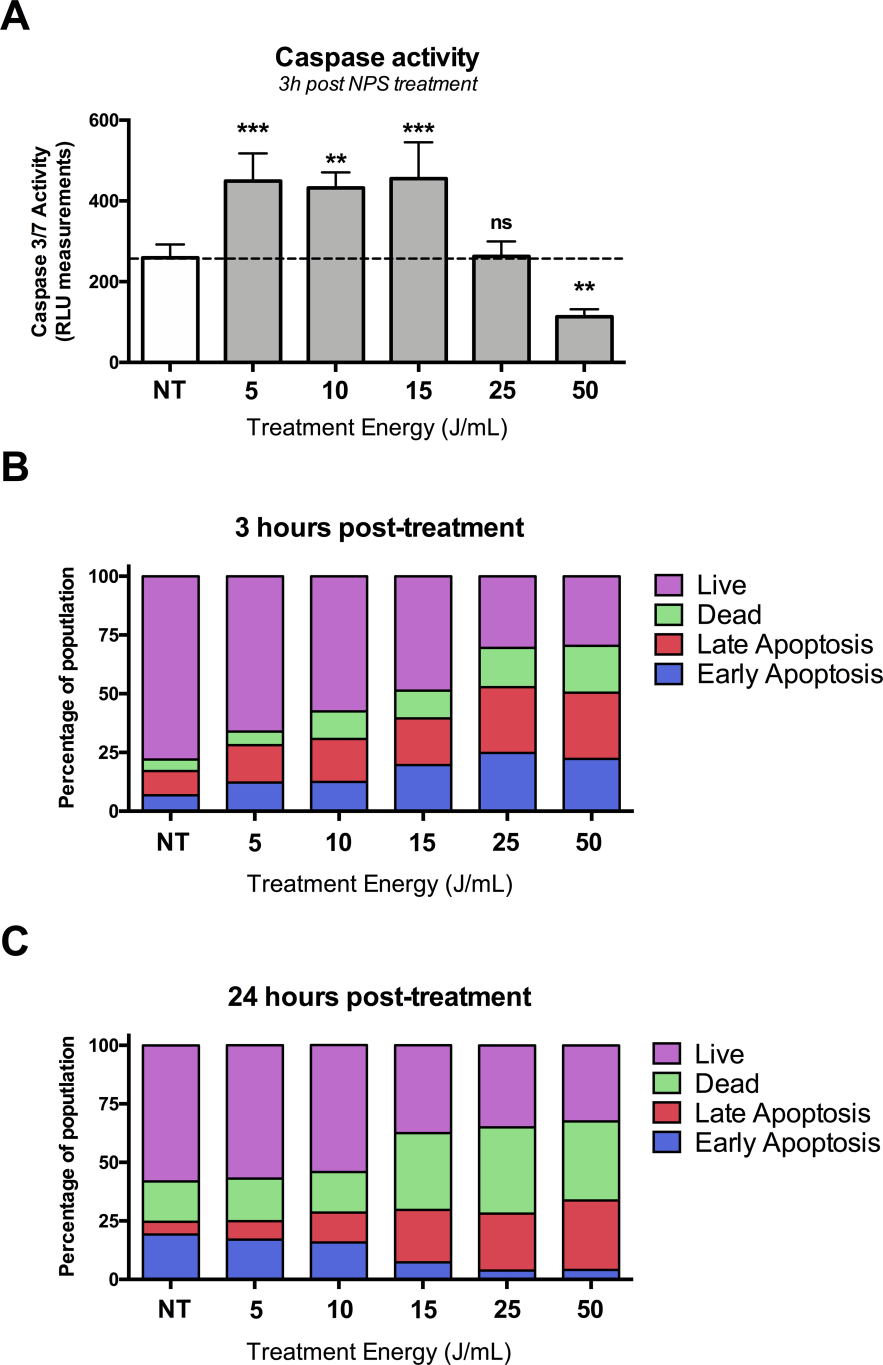
NPS treatment of C3.43 cells results in significant upregulation of caspase 3/7 activity at lower treatment energies. **(A)** Measured levels of activated caspase 3/7 in cells at 3h post NPS treatment for a range of NPS energy densities. Data shown as the mean of 4 experiments and the error bars represent the standard error of the mean (**p<0.01 ***p<0.001, One-way ANOVA followed by Dunnett’s multiple comparisons test to untreated cells). **(B)** Mean distribution of treated C3.43 tumor cells in early and late apoptosis at 3 h post-treatment with indicated NPS energy density **(C)** Data collected 24 h post NPS treatment.

NPS has been shown to trigger immunogenic cell death in many cell lines as indicated by the release of danger-associated molecular pattern molecules, caspase 3 activation, and phosphatidylserine translocation [18, 19]. As an initial indicator of whether NPS was an appropriate modality of treatment to induce an immunogenic cell death response in the C3.43 cell line, we sought to measure the levels of caspase activity and optimize the treatment conditions so they resulted in an immunogenic cell death profile within the target cell line. C3.43 cells were treated *in vitro* with NPS by placing the cells in an electroporation cuvette and pulsing them with a range of pulse numbers using 15 kV/cm, 100 ns-long pulses at 2 pps. We then measured the amount of activated caspase 3/7 1h, 3h and 24 h after NPS treatment. At 3h post treatment, we found a statistically significant increase within the 5, 10, and 15 J/ml treatment groups (p<0.01, One-way ANOVA followed by Dunnett’s multiple comparisons test against the untreated group) (**Fig 2A**) [18]. Interestingly, there was a significantly lower level of measurable caspase 3/7 activity at 50 J/mL (p<0.01, comparison to the non-treated group), which suggests that in order to induce an immunogenic cell death profile it is important to control the amount of energy applied to the target cells.

As a second measure of apoptosis and to profile the cell populations post treatment, we analyzed NPS-treated C3.43 cells using Annexin V staining by flow cytometry (**Fig 2B and 2C**). Within one hour, about 50% of the cells treated with 15–50 J/ml were in early or late apoptosis compared to only 17% of untreated cells. This percentage did not change at 3 h, but there was a measurable increase in the number of dead cells at both 3 and 24 h (3h data not shown) (**Fig 2B and 2C**). This is the expected progression from apoptosis to necrosis following NPS treatment.

### NPS treatment is able to clear established C3.43 tumors in immune-competent mice, eliminating or significantly reducing tumor volume and improving survival

**Fig 3:**
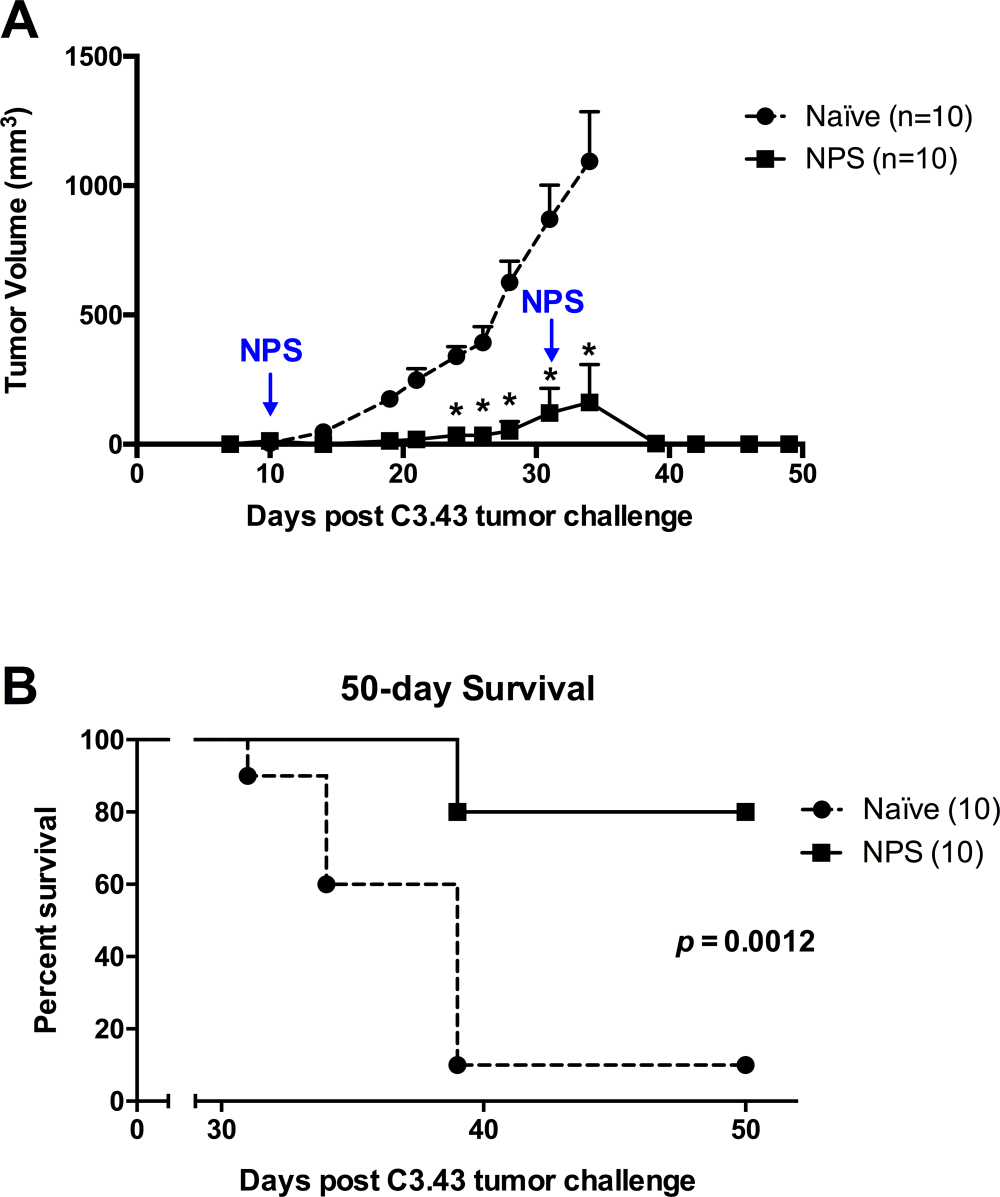
NPS treatment of primary tumors results in significant levels of tumor clearance, enhanced survival, and is effective during multiple applications. Groups of 10 mice were s.c. challenged with C3.43 tumors. 10-days post tumor challenge mice were given NPS (3 pps, 30kV/cm) treatment at the tumor site. Mice with recurring tumors received a second treatment on day 31 **(A)** Mean tumor volume (±SEM) of untreated and NPS treated mice (*p<0.05, unpaired students t-test at each time point). Volume measurements of untreated group displayed until there was a loss of 3 or more mice within the group due to euthanasia endpoints met **(B)** 50-day survival curve of groups with no treatment (naïve, median survival 39 days) or NPS treatment (NPS) (p<0.0012, Mantel-Cox Log Rank test). Data are representative of 2 independent experiments.

NPS has been used previously to treat non-viral transformed tumors with the finding that calibrated treatments can cause complete tumor ablation shortly after NPS application, induce an anti-tumor immune response that eliminates secondary tumors, and generates immunological memory that prevents successful tumor re-challenge [13, 20, 21]. To test whether NPS was an appropriate modality of treatment for established HPV-transformed tumors, we challenged groups of 15 immuno-competent mice with the C3.43 cell line (s.c. 1x10^5^ cells) and allowed them to grow for 10 days to ~50 mm^3^ in volume. NPS was applied and mean tumor volume and overall survival was recorded in the mice that were not used for ELISpot experiments. Over the next several days, tumors began to shrink and by two weeks post-treatment, most mice exhibited only a small scab where the original tumor was growing or had skin that healed completely (**Fig 1E**). Over half of the mice were able to clear tumors within one-week of treatment and mean tumor volumes were significantly lower in the NPS-treated group beginning at day 14 post treatment (day 24 post tumor challenge) and remained significant for the duration of the experiment (p<0.05, unpaired students t-test) **(Fig 3A)**. Overall survival of the NPS-treated mice was significantly improved (**Fig 3B**) (p<0.0001, Mantel-Cox Log Rank test). If primary application of NPS did not cause complete tumor regression, as was seen in 5 mice (with one additional mouse harboring a tumor too large for treatment), we were able to give a second, identical treatment. This second application appeared to be just as effective at eliminating remaining tumors. This finding suggests that the C3.43 tumors that were not cleared by the first NPS treatment did not develop any resistance to treatment, and, therefore, NPS therapy may be used more than once against recurrent tumors if initial treatments do not result in complete regression.

### NPS treatment generates effector CD8^+^ T cells that respond to unidentified antigens in the C3.43 model and enhances lymphocyte infiltration into tumors

**Fig 4:**
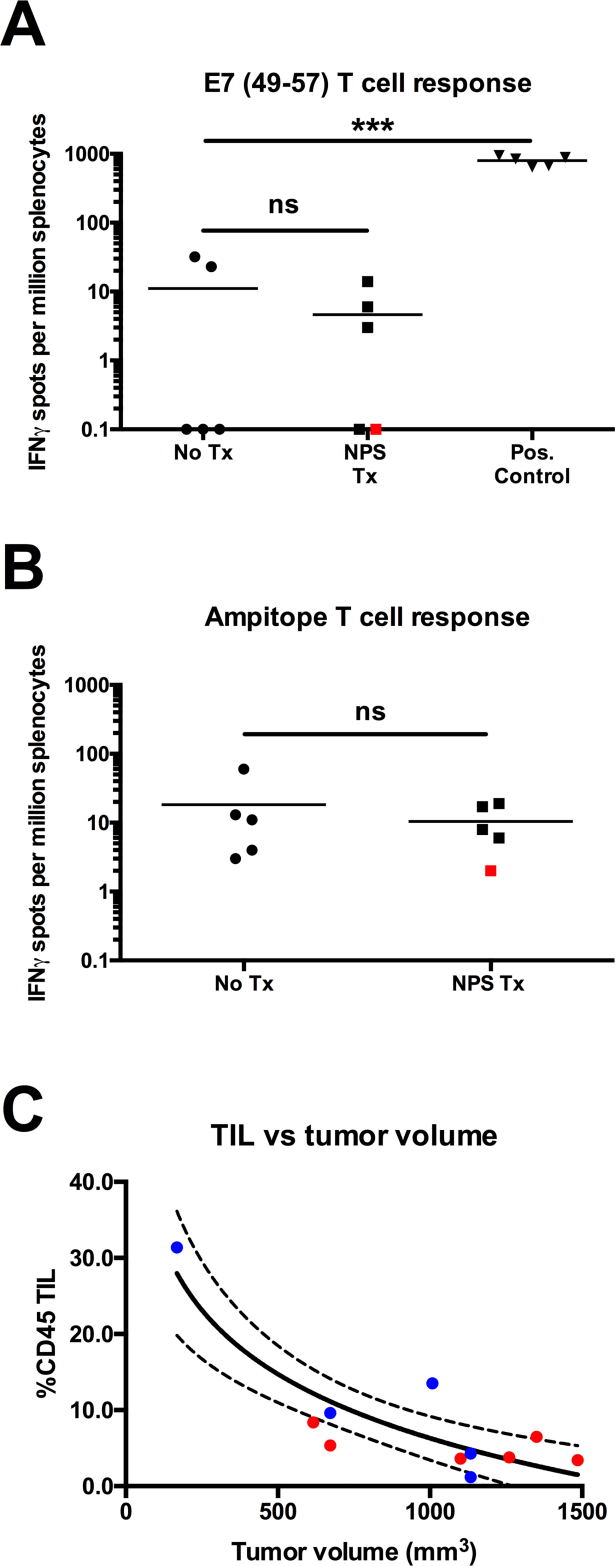
NPS-treated mice lack splenocytes recognizing known tumor antigens, but show an inverse correlation between TIL vs tumor volume. Groups of 5 mice were either untreated or treated with NPS therapy, followed by analysis for tumor antigen-specific T cells by IFNγ ELISpot assay. **(A)** Number of E7_(49–57)_-specific IFN-γ producing splenocytes were measured from mice receiving indicated treatments (ns = p>0.05, ***p<0.001, one-way ANOVA followed by Tukey’s multiple comparison test, comparisons to the naïve group shown). Black squares indicate mice that had cleared tumors post NPS treatment. Red square indicates a mouse with a recurrent tumor. Positive control mice were vaccinated with a viral-vector E7 based vaccine in the absence of tumor. **(B)** Number of ampitope-specific IFN-γ producing splenocytes measured from mice receiving indicated treatments (ns = p>0.05, unpaired students t-test). **(C)** Nonlinear regression analysis of tumor volume (x-axis) plotted against %CD45+ TILs detected via flow cytometry (R^2^ = 0.79, p = 0.0003 with the null hypothesis that slope = 0; 95% confidence intervals shown). Blue dots correspond to mice receiving NPS treatment that did not clear primary tumors. Red dots correspond to mice that had tumors which grew during re-challenge events. ELISpot data are representative of two independent experiments.

Two of the main challenges in immunotherapeutic approaches to treating solid-tumors are: 1) being able to generate an effective immunogenic anti-tumor response; and 2) enhancing the number of immune effector cells found within the tumor microenvironment. Because our *in vitro* data suggested immunogenic cell death could be induced in C3.43 cells and we saw partial inhibition of re-challenge tumor formation *in vivo*, we wanted to determine if NPS treatment was inducing CD8+ T cells to known tumor antigens and whether NPS treatment resulted in a change in the number of lymphocytes present within the tumor tissue. As in our initial challenge experiments, we applied NPS treatment (3 pps, 30kV/cm, 70 A) to subcutaneous C3.43 tumors of approximately 50 mm^3^ (10-days post challenge). Splenocytes were isolated from both tumor-bearing naïve mice and mice that had undergone NPS-treatment of primary tumors at day 34 post-tumor challenge. Splenocytes were incubated with an E7_(49–57)_ peptide associated with anti-HPV16 T cell responses in B6 mice as well as a cryptic epitope from the ampicillin resistance gene (ampitope) that is known to be expressed on the C3.43 cell line [22]. NPS-treated mice showed no reactivity to either of these peptides (**Fig 4A and 4B**) (p>0.05, unpaired students t-test), suggesting that if there is an immune response against the C3.43 cell line, it is against an unidentified antigen(s). As a control, splenocytes collected from age-matched, non-tumor challenged mice given the VRP positive control vaccine showed significant reactivity to the E7_(49–57)_ peptide.

To assess the effects of NPS on leukocyte infiltration in tumor tissue, percentage of cells expressing the pan-leukocyte marker CD45^+^ isolated from tumor tissues were plotted against tumor volumes for mice from ELISpot and survival experiments. Nonlinear regression analysis showed that there was an inverse correlative relationship between the size of the tumor and the percentage of CD45^+^ TILs found within isolates (**Fig 4C**) (p<0.0003 with the null hypothesis that slope = 0, R^2^ = 0.79). This suggests that mice having smaller tumors post NPS treatment have greater number of infiltrating lymphocytes present, contributing to the reduced growth rate and tumor volumes. Increased immune effector cells present within the tumor microenvironment may contribute to generating an anti-tumor response to novel antigens presented during immunogenic cell death of the C3.43 tumors.

### CD8 depletion in mice with NPS-treated primary tumors results in loss of protection against tumor re-challenge

**Fig 5:**
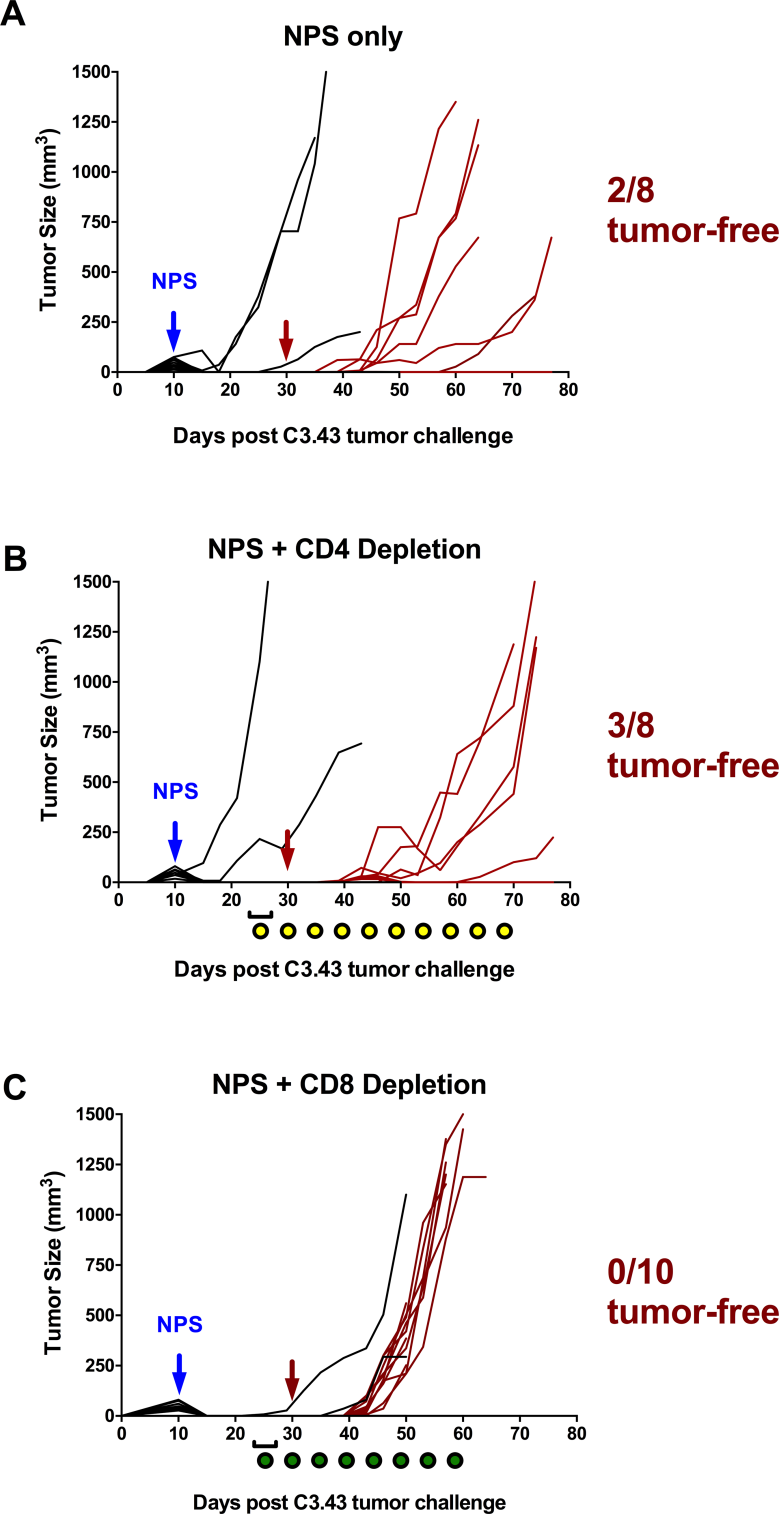
NPS treatment of tumors results in a CD8-dependent adaptive immune response. Shown are the individual tumor growth profiles of primary and rechallenge events in mice receiving NPS with or without selective depletion. Growth curves of primary (black) and re-challenge tumors (red) of NPS-treated mice with or without selective depletion of CD4 or CD8 T cells are displayed. **(A)** Mice received NPS treatment of primary tumor only (3 pps, 30 kV/cm, 70 A). **(B and C)** Mice received NPS treatment of primary tumor (3 pps, 30 kV/cm, 70A) followed by selective depletion of CD4 cells **(B)** or CD8 cells **(C)** with the administration of either an αCD4 mAb (yellow dots) or αCD8 mAb (green dots. The red arrows indicate the day of tumor re-challenge.

Effector CD8+ T cells that are produced during an immunogenic response to tumors are critical when it comes to effective immunotherapy resulting in tumor clearance, and have been shown to be a prognostic indicator of disease outcome [23, 24]. Given that after NPS treatment of the C3.43 tumor cells we found an increase in lymphocytes present within tumors but were not able to identify CD8+ T cells that responded to known antigens, we next examined whether selective depletion of CD8+ or CD4+ cells would result in changes in the growth rate of re-challenge tumors. Mice bearing ~30 mm^3^ tumors were treated with NPS and then randomized into three groups of 10. Primary tumor regression was observed in the majority of mice (**Fig 5**), as was shown previously. Starting three days prior to tumor re-challenge two of the NPS-treated groups were CD4- or CD8-depleted by intra-peritoneal administration of daily doses of anti-CD4 or anti-CD8 antibody. Depletion of specific populations was verified by flow cytometry from blood collected via retro-orbital bleeds and verified on a bi-weekly basis during maintenance dosing of depletion antibodies. Tumor re-challenge (s.c. 1x10^5^ cells) was carried out on all three groups as well as a fourth group of age-matched naïve mice. Tumor growth and overall survival was monitored. Individual growth curves for both the primary NPS-treated tumors (black) and the re-challenge tumors (red) show that NPS-treated control and NPS-treated CD4-depleted mice (**Fig 5A and 5B**) were protected against tumor re-challenge or tumor growth was delayed, indicative of an ongoing anti-tumor immune response, while NPS-treated CD8-depleted mice (**Fig 5C**) have a tumor growth and survival profile similar to the naïve group.

**Fig 6:**
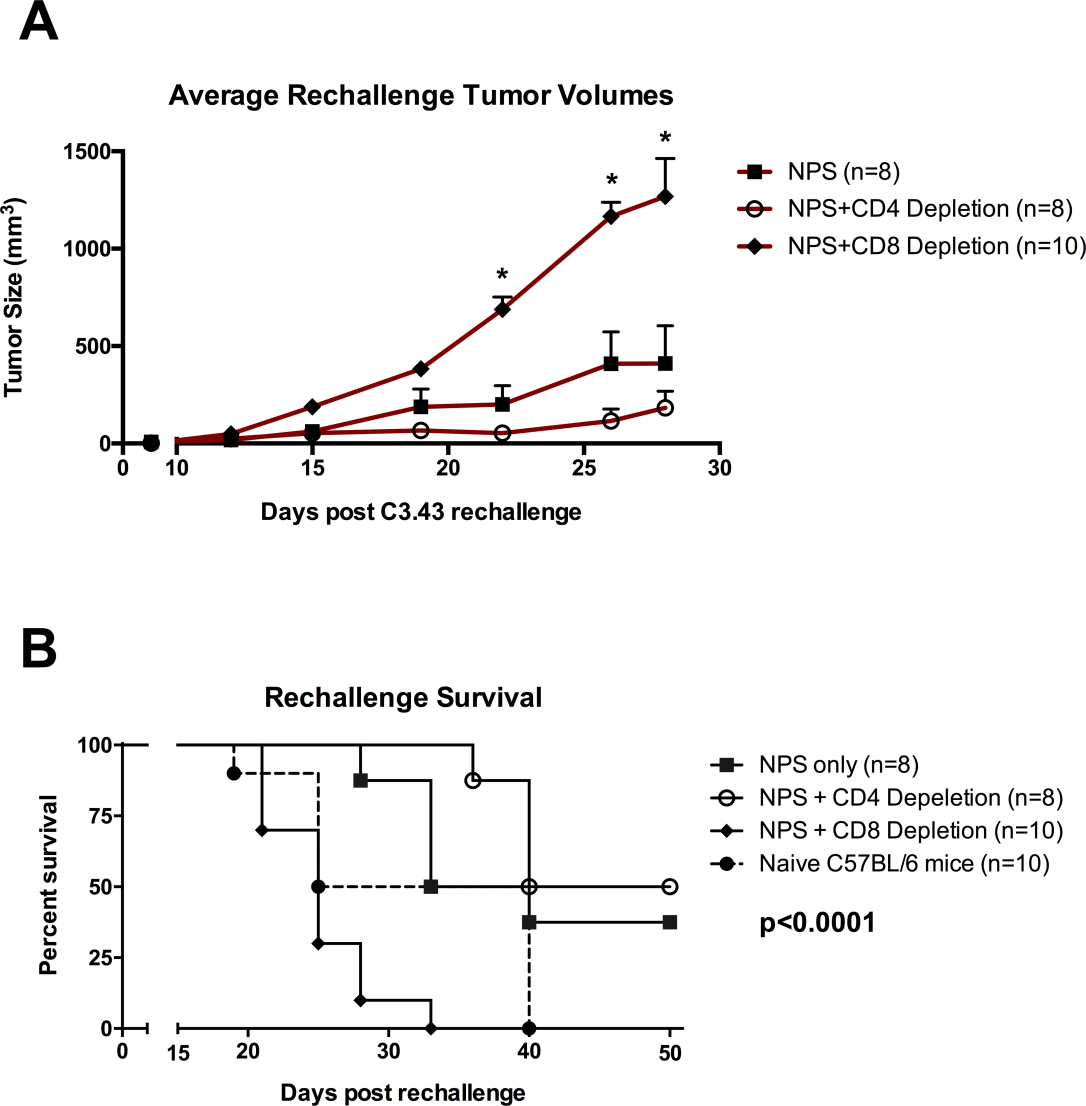
NPS treatment of primary tumors results in reduction in tumor rechallenge growth rate or protection against rechallenge, all dependent on presence of CD8 T cells. Mice that had cleared tumors in initial NPS treatment (3 pps, 30kV/cm, 70 A) were re-challenged s.c. by injecting with C3.43 tumor cells on the contralateral side. Prior to the re-challenge, indicated groups were dosed with anti-CD4 or anti-CD8 antibodies three days prior to tumor challenge followed by maintenance dosing every 3^rd^ day. **(A)** Mean tumor volumes of mice post tumor-rechallenge (+ SEM) (*p<0.05, One-way ANOVA followed by Dunnett’s multiple comparison test to the NPS treated groups). **(B)** Survival curve of treatment groups, including an age-matched naïve group (closed circle with dotted line) (p<0.0001, Mantel-Cox Log-rank test). The percentage of the animals in each treatment group that were still alive is plotted for up to 50 days post tumor rechallenge.

When comparing mean tumor volumes in re-challenged mice, CD8-depleted mice displayed significantly larger tumors beginning on day 22 post-tumor rechallenge (p<0.05, one-way ANOVA followed by Tukey’s multiple comparisons test) (**Fig 6A**). This finding suggests that specific elimination of CD8+ cells directly eliminates any protective immunity induced by NPS treatment within all mice in the group. Overall survival analysis of re-challenged mice showed a significant difference between groups (p<0.001, Mantel-Cox log-rank test) with median survival of CD4-depleted mice (45 days) and NPS-treated mice (36.5 days) higher than CD8-depleted or naïve challenged mice (25 and 32.5 days, respectively) (**Fig 6B**), suggesting that NPS-driven improvements in survival are contingent upon the presence of CD8+ cells.

## Discussion

HPV-associated cancers found in the anogenital and oral mucosa are an increasing global health burden. Several conventional therapies have been used to ablate HPV-associated cancers, including Loop electrosurgical excision [25], radiotherapy [26], Mohs surgery [27] and cryotherapy [28, 29], however efficacies vary from 23% to 94% and there remains a significant level of recurrence [28]. None of these ablative therapies to date have been shown to induce an immunogenic response against targeted tumors, which may be an underlying explanation for the rates of disease recurrence. The current landscape of cancer immunotherapy relies on eliciting or enhancing immune responses to tumor-associated antigens (TAA), which can be in the form of viral proteins or germline mutations, that are found in primary and metastatic cancer sites with the goal of eliminating these targets and providing immunological memory against the TAAs associated with the cancer. In line with these goals, we show for the first time that NPS therapy is able to eliminate primary HPV16+ tumors in mice, enhance lymphocyte infiltration into tumor tissue, and also induce a CD8-dependent protection against HPV16+ tumor rechallenge.

It has been reported that NPS treatment of non-viral-transformed tumor cells results in the release of the three classical danger-associated molecular pattern molecules: calreticulin, ATP, and HMGB1 [18], all associated with programmed cell death. Many of the steps in this pathway have been identified: NPS has been shown to generate transient nanopores in the plasma membrane and organelle membranes of treated tumor cells that allow the movement of ions across these membranes [16, 30]. An immediate consequence of this is an increase in cytoplasmic Ca^2+^ and the permeabilization of the endoplasmic reticulum (ER) and mitochondria [31, 32]. The spike in cytoplasmic Ca^2+^ has been found to stimulate reactive oxygen species generation (ROS) [33] and to trigger apoptosis when a sufficient number of spikes were generated. Simultaneously, ER-permeabilization stresses this organelle, and combined with ROS can lead to the translocation of the ER protein, calreticulin, to the plasma membrane where it initiates an “eat me” signal to dendritic cells [34]. Upon processing tumor proteins, the dendritic cells may then present tumor neo-antigens to the immune system and generate specific CD8^+^ cytotoxic T cells that will circulate in the body to seek out tumor cells expressing these novel targets [13]. With our data, we not only see an activation of these immunogenic cell death (ICD) pathways *in vitro*, but also promising results *in vivo* that serve as evidence for immunological responses against the tumors.

The model we have used in this study, C3.43, has viral protein expression driven by the homologous HPV promotor [14]. As a result of this C3.43 tumors have a lower abundance of HPV oncogenic proteins and more closely resembles the immunogenicity arising in HPV-transformed tumors that are seen clinically. Immuno-genomic approaches have shown that successful immunotherapy using adoptive transfer to treat HPV+ primary tumors and metastatic events in patients is driven by T cells recognizing immuno-dominant mutated cancer germline neo-antigens rather than expected targets such as E6 or E7 viral proteins [35], which we believe resembles the response being created by our NPS treatment *in vivo*. Specifically, NPS treated mice do not show T cell reactivity to either the HPV16 E7_49-57_ peptide nor the ampitope sequence, however depletion of CD8+ cells and subsequent loss of protection against tumor challenge indicate that NPS is inducing an immune response against the tumor driven by one or more neo-antigens that have yet to be identified. It should also be acknowledged that natural killer (NK) cell populations are critical in preventing early HPV+ tumor growth and act to bolster immune responses against tumors [36], and that a subset of CD8+ NK cells may have played a role in the protective immunity induced by NPS treatments.

In addition to the induction of anti-tumor immunity, successful immunotherapy relies on homing and infiltration of effector cells into the tumor itself. Within our experiments we found that NPS therapy results in a physical disruption of tumor tissue as some levels of edema are seen shortly after treatment. We believe that because of this combination of disruption to the tumor architecture and induction of ICD, there is a greater opportunity for lymphocytes to traffic to the apoptotic tumor cells. The inverse correlation seen between tumor size and frequency of CD45+ TIL may be driving this response as antigen presenting cells within the tumor microenvironment are able to process all TAAs, viral and germline mutation based, found within the tumor cells undergoing ICD. In a similar manner, dendritic cell-based immunotherapies are shown to be more effective when cells are primed with tumor cell proteins rather than specific tumor antigens found in the targeted tumor as it believed to generate a multi-faceted effector response against target tumors (reviewed in [37]).

In summary, our findings demonstrate that NPS can be used to successfully treat viral-driven cancers. Specifically, we show NPS eliminates HPV16-transformed tumors with a single treatment lasting about 3 minutes, and multiple treatments can be administered in the case of suboptimal tumor regression. This modality of treatment has the potential to not only ablate primary tumors in patients, but also to generate an immune response against HPV+ tumors, ultimately resulting in the protection of individuals from tumor recurrence and potential elimination of metastatic events, an effect that has been previously reported in mice and dogs bearing non-viral driven tumors [19–21].

## Supporting information

S1 File Compiled excel datasheet for experiments with individual tumor measurements. Tabs on spreadsheet include individual experiment data used to generate figures.
(XLSX)

## Abbreviations

HPV: human papillomavirus
NPS: Nano-Pulse Stimulation
PBS: phosphate-buffered saline
IFNγ: interferon-gamma
RLU: raw luminescence unit
ICD: immunogenic cell death
PS: phosphatidylserine
mAb: monoclonal antibody
ELISpot: Enzyme-Linked ImmunoSpot
TIL: tumor-infiltrating lymphocytes
PCR: polymerase chain reaction
A: amp
ns: nanosecond
kV: kilovolt
J: joule
HBSS: Hanks buffered saline solution
pps: pulses per second
VRP: HPV16-Venezuelan Equine Encephalitis replicon particle
s.c.: subcutaneous
NK: natural killer
DC: dendritic cell
